# Supplementary material for: Time‑resolved multi-omic analysis of paclitaxel exposure in human iPSC‑derived sensory neurons unveils mechanisms of chemotherapy‑induced peripheral neuropathy
Source: Cell Death Dis. 2026 Feb 10;17(1):211. doi: 10.1038/s41419-026-08445-2 (PMC12921266; doi:10.1038/s41419-026-08445-2)
Supplement: Supplementary file 15 — Dataset 1. [file 41419_2026_8445_MOESM15_ESM.zip › CDD_Open_Data/04_RNA_Seq/Timeline_panel_plots_etc_2023-01-23_1609.zip!/Panel_Plots_etc/Evidence_Plots/post5d/evidence_plot_standard contrasts_msigdb_reactome_5d_PTX_vs_DMSO_ID6_M7455_REACTOME_INTRINSIC_PATHWAY_FOR_APOPTOSIS.pdf]

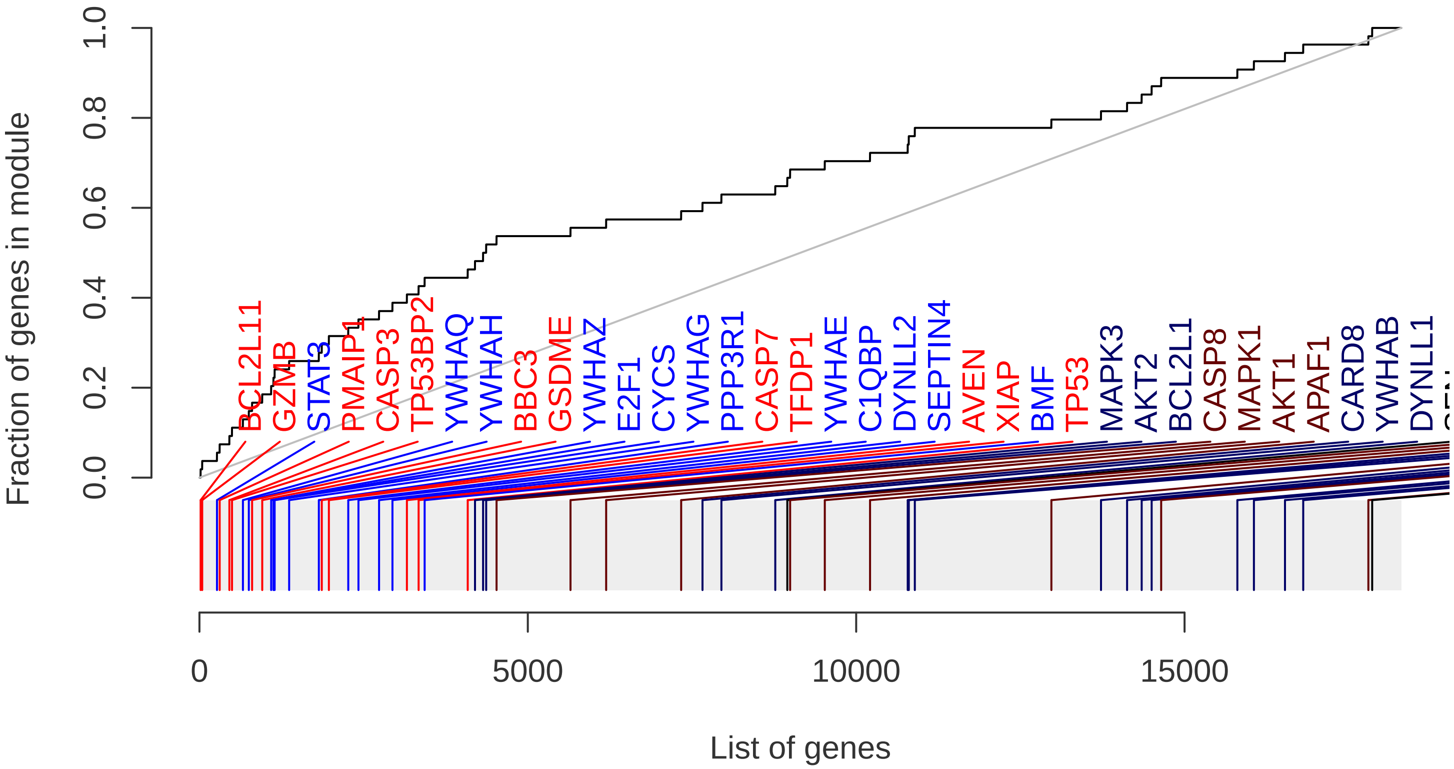

Fraction of genes in module
0.0
0.2
0.4
0.6
0.8
1.0
BCL2L11
GZMB
STAT3
PMAIP1
CASP3
TP53BP2
YWHAQ
YWHAH
BBC3
GSDME
YWHAZ
E2F1
CYCS
YWHAG
PPP3R1
CASP7
TFDP1
YWHAE
C1QBP
DYNLL2
SEPTIN4
AVEN
XIAP
BMF
TP53
MAPK3
AKT2
BCL2L1
CASP8
MAPK1
AKT1
APAF1
CARD8
YWHAB
DYNLL1
0
5000
10000
15000
List of genes
